# Development and validation of machine learning models for predicting functional outcome after low-dose alteplase in the extended time window for acute ischemic stroke

**DOI:** 10.3389/fnins.2026.1750031

**Published:** 2026-05-08

**Authors:** Huiru Chen, Qian Gui, Kangxiang Ji, Mengfan Ye, Jieji Zhao, Yan Kong, Guanhui Wu, Xin Tan

**Affiliations:** 1 Department of Neurology, The Affiliated Suzhou Hospital of Nanjing Medical University, Suzhou, Jiangsu, China; 2 Department of Neurology, The First Affiliated Hospital of Soochow University, Suzhou, Jiangsu, China; 3 Department of General Medicine, Shanghai Fifth People's Hospital of Fudan University, Shanghai, China; 4 Department of Occupational Disease, Shanghai Pulmonary Hospital of Tongji University, Shanghai, China

**Keywords:** 4.5–9 h, acute ischemic stroke, intravenous thrombolysis, low-dose alteplase, machine learning

## Abstract

**Background:**

This study aims to develop machine learning (ML) models to predict 90-day functional outcomes for acute ischemic stroke (AIS) patients receiving thrombolysis with low-dose alteplase at 0.6 mg/kg between 4.5 and 9 h after symptom onset.

**Methods:**

We conducted a retrospective analysis of AIS patients receiving thrombolysis between August 1, 2019 and August 31, 2023. Eligible patients were randomly divided into training and validation sets in a 7:3 ratio. Good functional prognosis at 90 days were defined as modified Rankin scale score (mRS) ≤2. Least Absolute Shrinkage and Selection Operator (LASSO) regression was used to select optimal features. Five ML algorithms were employed to construct prediction models. Model performance was evaluated using receiver operating characteristic (ROC) curves, area under the curve (AUC) value, decision curve analysis (DCA), and calibration curves. SHapley Additive exPlanations (SHAP) plot was applied to interpret the model predictions.

**Results:**

A total of 202 patients were randomly divided into training (*n* = 142) and validation (*n* = 60) sets. The rate of poor functional prognosis at 90 days was 56.34% in the training set and 56.67% in the validation set. Random Forest (RF) model showed the best discriminative ability with the highest AUC of 0.854 in the validation set. Key predictive features included age, baseline systolic blood pressure, white blood cell count, baseline National Institutes of Health Stroke Scale (NIHSS) score, wake-up stroke, the absolute difference volume between the ischemic infarct and the penumbra, intracranial hemorrhage, hemorrhagic transformation classification, and occurrence of pneumonia.

**Conclusion:**

The RF-based ML model demonstrated clinical utility for post-intravenous thrombolysis risk stratification by identifying patients at higher risk of poor functional outcomes.

## 1 Introduction

Ischemic stroke remains a leading cause of disability and mortality (Powers, 2020). While current guidelines recommend intravenous thrombolysis (IVT) as the standard treatment for acute ischemic stroke (AIS) within 4.5 h of symptom onset, approximately 65%−75% of patients present beyond the therapeutic time window or with unclear onset times, rendering them ineligible for IVT and leading to poorer functional outcomes (Powers et al., 2019; Ma et al., 2019). Advances in neuroimaging and randomized controlled trials have extended the therapeutic time window of AIS. The WAKE-UP trial demonstrated the safety and efficacy of IVT for wake-up stroke (WUS) patients selected by a mismatch between diffusion-weighted imaging (DWI) and fluid-attenuated inversion recovery (FLAIR) (Thomalla et al., 2018). Subsequently, the EXTEND trial (Extending the Time for Thrombolysis in Emergency Neurological Deficits) further demonstrated that for AIS patients with salvageable brain tissue between 4.5 and 9 h after the onset could benefit from IVT, the onset of stroke was estimated as the midpoint of sleep for patients awoke with stroke symptoms (Ma et al., 2019). Despite an increased risk of symptomatic intracerebral hemorrhage, a significantly higher proportion of patients receiving IVT achieved minimal or no neurologic deficits (Ma et al., 2019). Consequently, the 2021 European Stroke Organization (ESO) guidelines recommend IVT with alteplase for selected AIS patients 4.5–9 h after onset when MRI or CT perfusion (CTP) confirms salvageable tissue and endovascular therapy is not planned (Berge et al., 2021). Multimodal MRI or CTP can help extend the IVT time window, making approximately 30% of WUS patients eligible for treatment and is associated with favorable clinical outcomes (Broocks et al., 2023; Nagai et al., 2017). The optimal dose of alteplase (standard vs. lower) within the extended time window remains a subject of clinical debate. The Enhanced Controls of Hypertension and Thrombolysis Stroke Study (ENCHANTED) demonstrated that low-dose alteplase (0.6 mg/kg) had a comparable safety and efficacy profile compared with the standard dose (0.9 mg/kg), although it did not reach its primary endpoint of non-inferiority criteria (Anderson et al., 2016). Given the higher risk of symptomatic intracranial hemorrhage in Asian populations compared to other ethnic groups following standard-dose alteplase, low-dose alteplase beyond 4.5 h time windows have been more widely adopted (Mehta et al., 2014).

The clinical outcomes of AIS receiving IVT demonstrate significant variability, with not all patients achieving a favorable prognosis. Studies indicate that even among patients with large vessel occlusion receiving endovascular therapy, 29%−67% still experience poor outcomes (Le Guennec et al., 2020). Key prognostic factors include patient age, atrial fibrillation, changes in the National Institutes of Health Stroke Scales (NIHSS) scores at baseline and post-thrombolysis, blood pressure levels, modified Trial of ORG10172 in Acute Stroke Treatment (TOAST) classification, infarct volume, and post-procedural complications (Le Bouc et al., 2018; Groot et al., 2020). To date, real-world clinical cohort studies on CTP guided IVT for AIS patients beyond the standard therapeutic windows remain limited. Given that delayed IVT in these patients may be associated with higher risks of intracranial hemorrhage (ICH) transformation and poor functional outcomes, developing prognostic models and analyzing related risk factors are urgent and crucial.

Recent advances in artificial intelligence and big data analytics have enabled widespread adoption of machine learning (ML) algorithms for the diagnosis of ischemic stroke (Murray et al., 2020). Conventional multivariate statistical methods are limited in processing complex, high-dimensional medical data, as they struggle to capture complex nonlinear interactions among variables. Moreover, with numerous predictive variables and multicollinearity, these models are prone to overfitting, which constitutes a significant methodological gap in existing predictive models (Jordan and Mitchell, 2015; Shehab et al., 2022). Imaging analysis provides direct anatomical and functional insights and enables quantification of stroke-related risk factors, such as the TOAST classification (Adams et al., 1993). By integrating imaging features with patients' clinical data, ML can further improve the prediction of treatment response and clinical outcomes (Zhao et al., 2024). Therefore, we conducted a retrospective analysis based on real-world clinical data from our center, aiming to develop prognostic prediction models for ischemic stroke patients treated beyond the time window with low-dose alteplase (0.6 mg/kg). SHapley Additive exPlanations (SHAP) plot was used to interpret the optimal model by visualizing feature importance, thereby facilitating early identification of key risk factors and supporting timely interventions to improve clinical prognosis.

## 2 Materials and methods

This study retrospectively enrolled AIS patients who received low-dose alteplase at 0.6 mg/kg through the emergency stroke green channel between August 1, 2019 and August 31, 2023. The patients were randomly allocated into a training set and a validation set at a ratio of 7:3. This study was approved by the ethics committee of Soochow University (Approval Number: 2024239).

### 2.1 Study population

Patients were eligible for inclusion if they met all of the following criteria: (1) Age >18 years; (2) Presenting within 4.5–09 h after symptom onset. For WUS or patients with unknown onset time, the onset of stroke was estimated as the midpoint between the last known to be well time and symptom recognition time; (3) Baseline NIHSS score 5–26; (4) CTP imaging following EXTEND trial (Ma et al., 2019): a mismatch ratio between the volume of ischemic penumbra and core infarct volume >1.2, an absolute volume difference >10 ml, and an infarct core volume <70 ml. CTP images were analyzed using the MIStar automated software (Loygue et al., 1981). The core infarct volume was defined as brain tissue with a relative reduction in cerebral blood flow of <30%, and the ischemic penumbra volume was defined as tissue with a delay time >3 seconds. (5) Provided written informed consent for alteplase thrombolysis. Patients were excluded if they had: (1) Pre-stroke modified Rankin Scale (mRS) score > 2; (2) Missing baseline or follow-up clinical records.

### 2.2 Data collection

Patient clinical and demographic characteristics were collected, including: (1) demographic factors: age and sex; (2) risk factors: hypertension, diabetes mellitus, hyperlipidemia, atrial fibrillation, previous stroke, antiplatelet or anticoagulant therapy; (3) clinical characteristics: baseline systolic/diastolic blood pressure (SBP/DBP), admission NIHSS score, WUS, stroke onset-to-door time (ODT), door-to-needle time (DNT), mechanical thrombectomy; (4) laboratory tests: fasting blood glucose (FBG), hemoglobin A1c (HbA1c), white blood cell count (WBC), C-reactive protein (CRP), platelet count (PLT), hemoglobin (HB), prothrombin time (PT), activated partial thromboplastin time (APTT), total cholesterol (TC), triglycerides (TG), low-density lipoprotein cholesterol (LDL-C), high-density lipoprotein cholesterol (HDL-C), homocysteine (HCY), blood urea nitrogen (BUN), serum creatinine (SCr); (5) Admission Imaging: Alberta Stroke Program Early CT Score (ASPECTS), core infarct volume, ischemic penumbra volume, and mismatch volume; (6) MRI-determined infarct-associated culprit vessels: anterior cerebral artery, middle cerebral artery, posterior cerebral artery, vertebral-basilar artery, internal cerebral artery; (7) TOAST classification: large-artery atherosclerosis, cardioembolism, small-artery occlusion, stroke of other determined etiology, stroke of undetermined etiology; (8) Safety Outcomes: any ICH (Hacke et al., 1998), hemorrhage transformation [European Cooperative Acute Stroke Study (ECASS-2)], symptomatic ICH (Wahlgren et al., 2007) and pneumonia (Smith et al., 2015). The summary of patient characteristics and variables is presented in Supplementary Table S1.

### 2.3 Outcome assessment

Prognosis assessment was conducted by two neurologists via telephone interview at the 90 days post-discharge. In case of disagreement, a third senior neurologist was consulted for final adjudication. Good functional prognosis was defined as mRS score ≤ 2, and poor functional prognosis was defined as mRS score > 2.

## 3 Feature selection

Given the large number of candidate predictors, limited sample size, and the presence of multicollinearity, we employed the Least Absolute Shrinkage and Selection Operator (LASSO) regression model for feature selection to improve model stability and reduce overfitting. The optimal regularization parameter Lambda (λ) was determined through 10-fold cross-validation to ensure model generalizability. The selected variables were then used to build a parsimonious and interpretable prediction model using backward stepwise logistic regression.

## 4 Machine learning model development

This study developed predictive models using five ML algorithmics: logistic regression, RF, XGBoost, SVM, and LightGBM. The optimal hyperparameter optimization were selected through 10-fold cross-validated grid search on the training set, with the area under the curve (AUC) serving as the primary optimization metric. The optimal probability threshold was determined by maximizing the Youden index on the validation set. Subsequently, model performance was evaluated on the validation set using confusion matrix-based metrics (positive predictive value, negative predictive value, and F1-score). Model performance was systematically evaluated across three key aspects: (1) discriminative ability using receiver operating characteristic (ROC) curve analysis, (2) calibration performance via calibration curves, and (3) clinical utility assessed by decision curve analysis (DCA). Model interpretability and visualizations were achieved through SHAP value decomposition, enabling both global feature importance quantification and individualized prediction explanations, with results visualized through summary plots and force diagrams.

## 5 Statistical analysis

Statistical analyses were performed using R version 4.3.1. Normally distributed continuous variables were presented as mean ± standard deviation (SD) and compared using the Student's *t*-test, while non-normally distributed variables were presented as median (interquartile range) compared using the Mann-Whitney *U* test. Categorical variables were presented as frequencies and compared using the Pearson Chi-squared or Fisher's exact tests. AUCs are reported with 95% confidence intervals estimated via the DeLong method. All statistical tests were two-sided, and a *P*-value < 0.05 was considered statistically significant.

## 6 Results

### 6.1 Demographic and clinical characteristics

**Figure 1:**
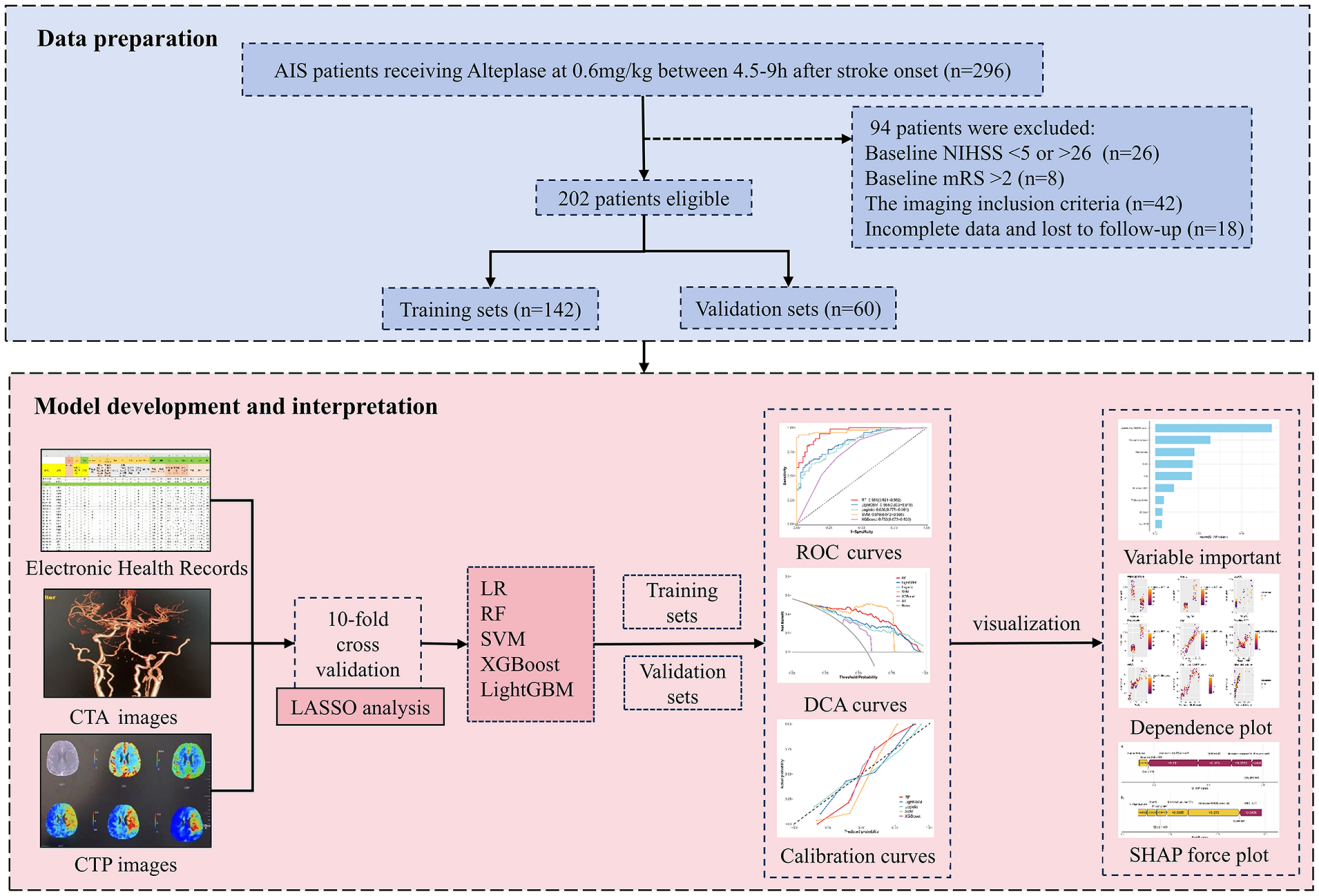
The Flow chart of this study design. AIS, acute ischemic stroke; NIHSS, National Institutes of Health Stroke Scale; mRS, modified rankin scale score; CTA, computed tomography angiography; CTP, computed tomography perfusion; LASSO, least absolute shrinkage and selection operator; LR, logistic regression; RF, random forest; SVM, support vector machine; XGBoost, extreme gradient boosting; LightGBM, light gradient boosting machine; ROC, receiver operating characteristic; DCA, decision curve analysis; SHAP, SHapley additive explanations.

A total of 202 patients were included in the study. The process of patient selection is illustrated in Figure 1. The median age of the cohort was 68 years (IQR 56.75–74.25), with 138 (68.3%) being male. All patients received low-dose alteplase intravenous thrombolysis therapy, with 73 patients (36.1%) undergoing bridging mechanical thrombectomy. The median ODT was 343.50 (IQR 288, 450.25) minutes, and the median DNT was 87 (IQR 69.75, 106) minutes. Among patients receiving IVT, the incidence of ICH was 21.3% (43/202), with approximately 27 cases (13.4%) demonstrating symptomatic hemorrhagic transformation. Pneumonia occurred in 75 cases (37.1%) during hospitalization. The dataset was randomly divided into training (*n* = 142) and validation (*n* = 60) sets at a 7:3 ratio, with poor-outcome proportions of 56.34% (80/142) and 56.67% (34/60), respectively. In the training set, the poor-outcome group showed significantly higher values in Age, Admission NIHSS scores, WBC, C-reactive protein, Blood urea nitrogen, Imaging parameters (ASPECT score, infarct core volume, ischemic penumbra volume, mismatch volume). Additionally, ICH incidence (25% vs 8.06%, p < 0.05) and pneumonia rates (51.25% vs 16.13%, p < 0.001) were significantly higher in the poor-outcome group. In the validation set, the poor-outcome group had significantly higher: prevalence of hypertension history, admission NIHSS scores, blood urea nitrogen levels, imaging parameters (ischemic penumbra volume and mismatch volume). This group also demonstrated significantly higher rates of hemorrhage transformation (ECASS-2 classification) and pneumonia (all p < 0.05). No other significant differences were observed in the remaining risk factors. Detailed clinical characteristics are presented in Table 1.

**Table 1:** The baseline characteristics of patients.

Variables	The training set (***n*** = 142)		P value	The validation set (***n*** = 60)		P value
	Poor outcome (*n* = 80)	Good outcome (*n* = 62)		Poor outcome (*n* = 34)	Good outcome (*n* = 26)	
Age (year)	71 (61.75, 76)	64.5 (54, 72)	0.005	66.21 ± 10.78	61.46 ± 13.08	0.140
Male, *n* (%)	54 (67.5)	42 (67.74)	>0.999	22 (64.71)	20 (76.92)	0.460
Previous history, *n* (%)						
Hypertension	60 (75)	38 (61.29)	0.117	26 (76.47)	12 (46.15)	0.032
Diabetes mellitus	31 (38.75)	19 (30.65)	0.409	13 (38.24)	7 (26.92)	0.519
Dyslipidemia	33 (41.25)	25 (40.32)	>0.999	13 (38.24)	14 (53.85)	0.346
Previous stroke	19 (23.75)	11 (17.74)	0.508	6 (17.65)	2 (7.69)	0.446
Atrial fibrillation	6 (7.5)	3 (4.84)	0.731	3 (8.82)	3 (11.54)	>0.999
Prior antiplatelet use	31 (38.75)	17 (27.42)	0.216	11 (32.35)	7 (26.92)	0.865
Prior anticoagulant use	12 (15)	11 (17.74)	0.833	4 (11.76)	4 (15.38)	0.717
Clinical features						
NIHSS score	15 (8.75, 19)	9 (6, 13)	<0.001	16.5 (12.25, 19.75)	7 (5, 10.75)	<0.001
Baseline SBP (mmHg)	156.52 ± 24.4	149.6 ± 24.76	0.098	157.82 ± 29.93	155.5 ± 20	0.720
Baseline DBP (mmHg)	89.58 ± 16	88.34 ± 15.36	0.641	88.5 ± 14.27	87.88 ± 14.7	0.871
Wake-up stroke	50 (62.5)	46 (74.19)	0.195	24 (70.59)	16 (61.54)	0.645
Onset-to-door time (min)	377.5 (288.75, 463.5)	344.5 (299.25, 444.25)	0.903	311 (284, 424.5)	307 (277.75, 390.25)	0.245
Door-to-needle time (min)	83 (68.75, 104)	89.5 (70.25, 107)	0.652	86.5 (74.25, 103)	87 (70.5, 108.25)	0.400
Mechanical thrombectomy, *n* (%)	29 (36.25)	22 (35.48)	>0.999	12 (35.29)	10 (38.46)	>0.999
Laboratory data						
FBG (mmol/L)	6.98 (6.4, 8.66)	6.77 (5.82, 8.42)	0.211	6.78 (5.61, 7.4)	6.64 (5.46, 8.11)	0.994
HbA1c (%)	6 (5.5, 6.43)	6 (5.6, 6.57)	0.806	6 (5.62, 6.5)	5.8 (5.5, 6.38)	0.241
WBC (×10^9^/L)	9.5 (7.6, 12)	7.97 (5.93, 10.51)	0.002	9.96 (6.87, 12.04)	7.81 (6.84, 9.97)	0.114
PLT (×10^9^/L)	199 (155.25, 237.25)	203 (157, 234.75)	0.946	193.12 ± 74.14	218.04 ± 64.25	0.169
CRP (mg/L)	7.12 (2.06, 18.95)	3.21 (1.15, 11.27)	0.007	5.26 (2.02, 17.89)	8.4 (2.22, 14.32)	>0.999
HB (×10^9^/L)	135.88 ± 18.45	135.6 ± 15.83	0.923	134 ± 12.74	139.54 ± 19.88	0.222
PT (s)	13.15 (12.17, 13.9)	12.9 (11.61, 13.6)	0.147	12.82 ± 1.44	12.21 ± 1.74	0.156
APTT (s)	31.55 (27.28, 34.5)	31 (25.75, 34.45)	0.419	28.6 (23.02, 33.62)	26.7 (23.6, 31.13)	0.840
BUN (mmol/L)	6 (4.7, 7.03)	5.12 (4.21, 6.3)	0.008	6.42 (5.24, 8.97)	5.25 (4.04, 5.88)	0.008
CRE (umol/L)	70 (57.83, 81)	66.3 (56.3, 76.5)	0.427	67.85 (57.08, 89.85)	68.5 (59.05, 86.22)	0.743
TC (mmol/L)	4.28 (3.71, 5.12)	4.14 (3.39, 4.75)	0.354	4.06 ± 1.39	4.38 ± 1.02	0.313
TG (mmol/L)	1.04 (0.81, 1.61)	1.18 (0.84, 1.65)	0.503	1.21 (0.91, 1.72)	1.61 (1.13, 2.19)	0.159
LDL-C (mmol/L)	2.19 (1.28, 2.9)	2.16 (1.14, 2.83)	0.690	2.51 ± 1.06	2.67 ± 1.1	0.583
HDL-C (mmol/L)	1.19 (0.91, 1.96)	1.15 (0.94, 1.55)	0.733	1.13 (1.01, 1.34)	1.06 (0.87, 1.39)	0.507
HCY (umol/L)	12.2 (9.8, 16.22)	11.65 (9.2, 13.52)	0.664	10.45 (8.3, 19.25)	10.4 (8.62, 13.78)	0.875
Imaging result						
ASPECT	8 (7, 10)	9 (8, 10)	0.032	9 (7, 10)	9 (7, 10)	0.468
Infarct core volume (ml)	8.38 (1, 28.5)	2 (0, 11.86)	0.026	13.3 (1.12, 33.75)	3 (1.7, 8.85)	0.176
Ischemic penumbra volume (ml)	120 (57.83, 182)	51.11 (26.27, 110.42)	<0.001	140.1 (76.09, 190)	58 (33.25, 120.6)	<0.001
Mismatch volume (ml)	108 (51.92, 154.25)	48.05 (25.25, 92.67)	<0.001	103.8 (58.26, 167.57)	53.2 (30.5, 104.75)	0.008
Vascular territory of infarction, *n* (%)			0.540			0.524
Anterior cerebral artery	11 (13.75)	8 (12.9)		5 (14.71)	3 (11.54)	
Middle cerebral artery	44 (55)	36 (58.06)		17 (50)	16 (61.54)	
Posterior cerebral artery	1 (1.25)	3 (4.84)		1 (2.94)	3 (11.54)	
Vertebral-basilar artery	10 (12.5)	9 (14.52)		3 (8.82)	1 (3.85)	
Internal cerebral artery	14 (17.5)	6 (9.68)		7 (20.59)	2 (7.69)	
TOAST classification, *n* (%)			0.404			0.521
Large-artery atherosclerosis	45 (56.25)	37 (59.68)		22 (64.71)	17 (65.38)	
Cardioembolism	23 (28.75)	12 (19.35)		8 (23.53)	7 (26.92)	
Small artery etiology	1 (1.25)	2 (3.23)		1 (2.94)	1 (3.85)	
Other determined etiology	7 (8.75)	4 (6.45)		3 (8.82)	0 (0)	
Undetermined etiology	4 (5)	7 (11.29)		0 (0)	1 (3.85)	
Safety outcomes, *n* (%)						
Intracranial hemorrhage	20 (25)	5 (8.06)	0.016	12 (35.29)	6 (23.08)	0.460
Hemorrhage transformation ECASS-2			0.198			<0.001
HI1	3 (3.75)	2 (3.23)		23 (67.65)	21 (80.77)	
HI2	5 (6.25)	0 (0)		1 (2.94)	0 (0)	
PH1	4 (5)	1 (1.61)		0 (0)	5 (19.23)	
PH2	6 (7.5)	3 (4.84)		3 (8.82)	0 (0)	
Symptomatic intracranial hemorrhage	14 (17.5)	5 (8.06)	0.165	5 (14.71)	3 (11.54)	>0.999
Pneumonia	41 (51.25)	10 (16.13)	<0.001	19 (55.88)	5 (19.23)	0.009

NIHSS, National Institutes of Health Stroke Scale; SBP, systolic blood pressure; DBP, diastolic blood pressure; FBG, fasting blood glucose; HbA1c, hemoglobin A1c; WBC, white blood cell count; PLT, platelet count; CRP, C-reactive protein; HB, hemoglobin; PT, prothrombin time; APTT, activated partial thromboplastin time; BUN, blood urea nitrogen; CRE, serum creatinine; TC, total cholesterol; TG, triglycerides; LDL-C, low-density lipoprotein cholesterol; HDL-C, high-density lipoprotein cholesterol; HCY, homocysteine; ASPECT, Alberta Stroke Program Early CT Score; TOAST, modified Trial of ORG10172 in Acute Stroke Treatment; ECASS: hemorrhage transformation (European Cooperative Acute Stroke Study-2 classification); HI, hemorrhagic infarction; PH, parenchymal hemorrhage.

### 6.2 Univariate and multivariate logistic regression analysis

Nine variables of the 41 variables were selected and later reserved as independent risk factors using the LASSO regression model with the “λmin (0.053)” criterion, which was achieved by 10-fold cross validation, to solve such multiple colinear relationships among the explanatory variables (Supplementary Figure S1). The final logistic model, including nine variables, included age, baseline SBP, WBC, admission NIHSS score, WUS, Mismatch volume, any ICH, ECASS-2 classification, and pneumonia.

### 6.3 Model development and performance comparison

**Figure 2:**
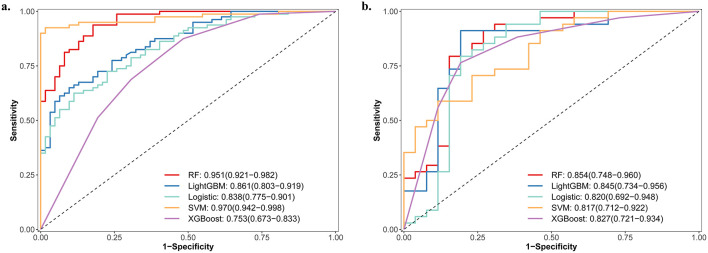
ROC curves of five ML models in the training **(a)** and validation **(b)** sets. ROC, receiver operating characteristic; RF, random forest; LightGBM, light gradient boosting machine; Logistic, logistic regression; SVM, support vector machine; XGBoost, extreme gradient boosting.

The feature subset selected by Lasso regression was subsequently incorporated into five ML models for comparative analysis. Confusion matrices are provided in Supplementary Figures S2–S6. Overall, the models showed good discrimination in both the training and validation sets, and detailed performance metrics are presented in Table 2 and Supplementary Table S2. The ROC cures for the training and validation sets are displayed in the Figures 2a, b. The RF model achieved the best performance in the validation set (AUC 0.854, 95% CI 0.748–0.960), followed by Light GBM (AUC 0.845, 95% CI 0.734–0.956), XGBoost (AUC 0.827, 95% CI 0.721–0.934), logistic regression (AUC: 0.820, 95% CI: 0.692–0.948) and SVM (AUC 0.817, 95% CI 0.712–0.922). To further assess internal stability and potential overfitting, we conducted internal cross-validation of the final RF model. The mean AUC was 0.797 ± 0.060 with five-fold cross-validation and 0.836 ± 0.091 with ten-fold cross-validation, and the corresponding ROC curves are shown in Supplementary Figure S7. The RF model achieved an accuracy of 0.783, sensitivity of 0.794, specificity of 0.769, positive predictive value of 0.818, negative predictive value of 0.741, and an F1-score of 0.806 in the validation cohort (Table 2). In the training cohort, the corresponding performance was accuracy 0.938, sensitivity 0.887, specificity 0.872, positive predictive value 0.872, negative predictive value 0.911, and F1-score 0.823 (Supplementary Table S2).

**Table 2:** Comparison of performance of machine learning and logistic regression in the validation cohort.

Models	AUC (95% CIs)	Sensitivity	Specificity	Accuracy	PPV	NPV	F1-score	Brier score
SVM	0.817 (0.712–0.922)	0.735	0.692	0.717	0.758	0.667	0.746	0.186
RF	0.854 (0.748–0.960)	0.794	0.769	0.783	0.818	0.741	0.806	0.160
Light GBM	0.845 (0.734–0.956)	0.559	0.885	0.700	0.864	0.605	0.679	0.155
Logistic regression	0.820 (0.692–0.948)	0.529	0.846	0.667	0.818	0.579	0.643	0.174
XGBoost	0.827 (0.721–0.934)	0.882	0.615	0.767	0.750	0.800	0.811	0.208

AUC, area under the curve; SVM, support vector machine; RF, random forest; Light GBM, light gradient boosting machine; XGBoost, extreme gradient boosting; PPV, positive predictive value; NPV, negative predictive value.

**Figure 3:**
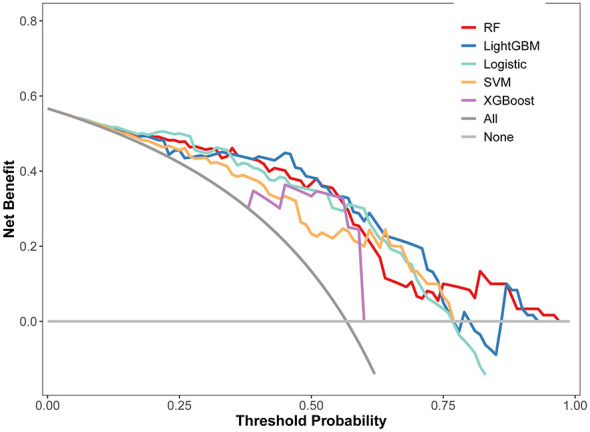
DCA curves of five ML models in validation sets. RF, random forest; LightGBM, light gradient boosting machine; Logistic, logistic regression; SVM, support vector machine; XGBoost, extreme gradient boosting.

The calibration curves indicated that the predictive values were highly consistent with the actual values, with Brier scores of 0.113 and 0.160 for the training and validation set in the RF model, respectively, as shown in Supplementary Figure S8. Figure 3 shows the DCA curves in the validation sets. demonstrating that when the high-risk threshold predicted by the RF model ranged from 10% to 70%, early intervention could provide substantial benefits (approximately 15–25%). Specifically, the maximum additional benefit reached 25% at the 10% threshold (equating to 25 fewer unnecessary interventions per 100 patients), with sustained gains of 15–20% at intermediate thresholds (50%). The DCA curves of the RF model in the training set were shown in Supplementary Figure S9.

### 6.4 Model explanation

**Figure 4:**
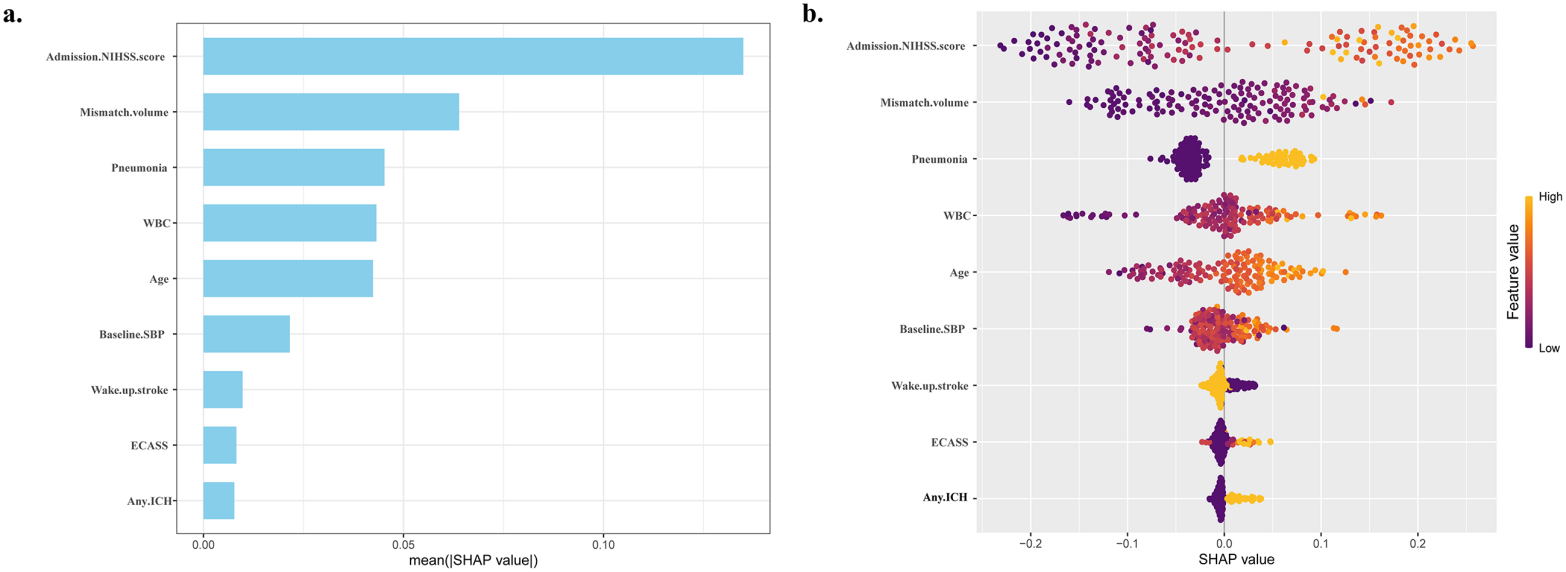
Global model explanation by the SHAP model. **(a)** SHAP summary bar plot; **(b)** SHAP summary dot plot. The X-axis represents the SHAP values, while the Y-axis displays the feature names arranged in descending order based on their contribution to the model. NIHSS, National Institutes of Health Stroke Scale; Mismatch volume, the difference between the ischemic penumbra volume and the core infarct volume; WBC, white blood cell count; ECASS, hemorrhage transformation (European Cooperative Acute Stroke Study-2 classification); ICH, intracranial hemorrhage.

**Figure 5:**
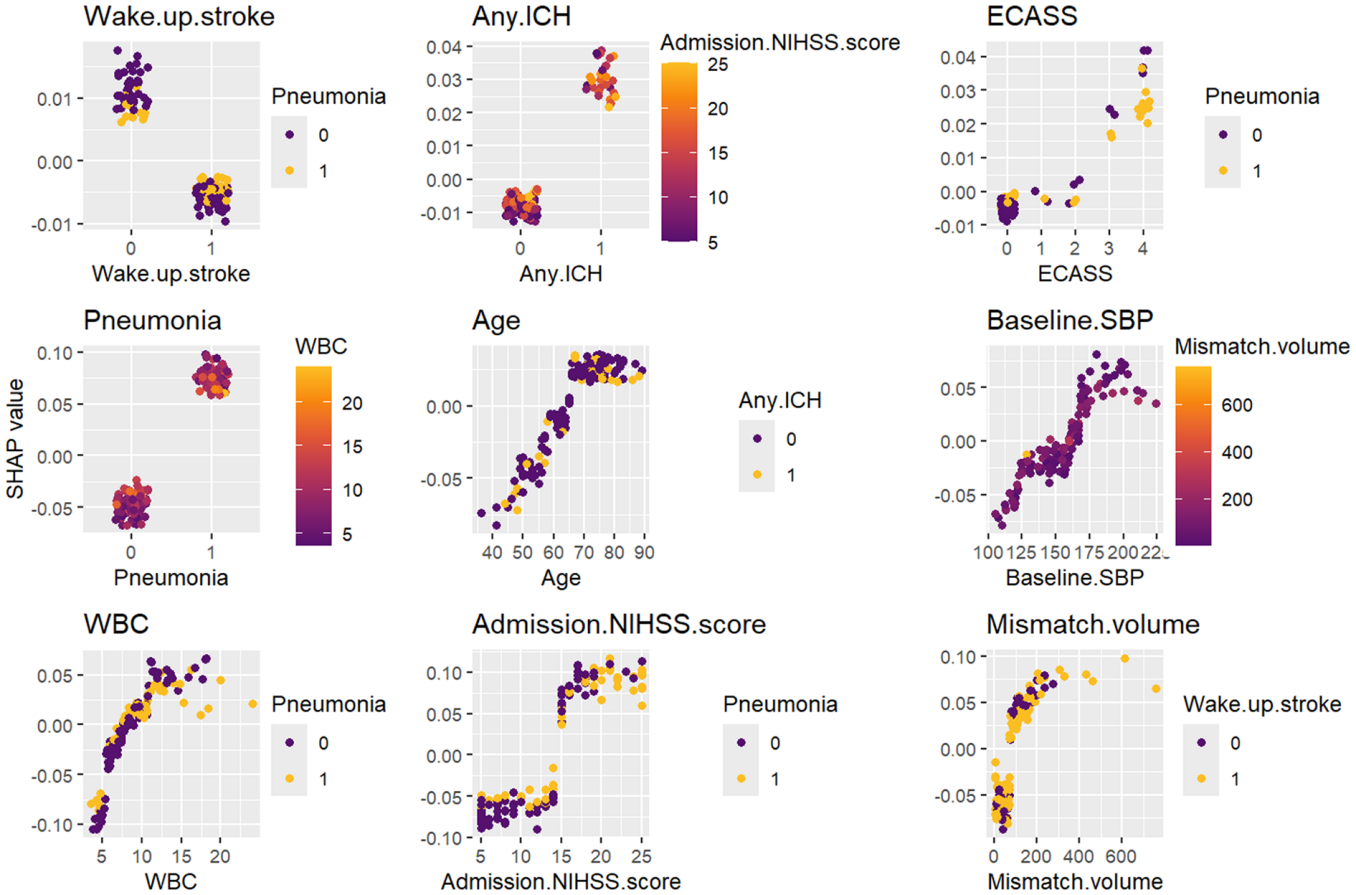
SHAP dependence plots for the RF model. These plots illustrate the influence of individual features on model's predictions, with each data point representing an individual patient. The vertical axis represents the SHAP value, while the horizontal axis corresponds to the actual value of the feature. For example, admission NIHSS ≥ 14 or mismatch volume ≥ 80 push the model's decision toward a poor clinical prognosis at 90 days. ICH, intracranial hemorrhage; ECASS, hemorrhage transformation (European Cooperative Acute Stroke Study-2 classification); SBP, systolic blood pressure; WBC, white blood cell count; NIHSS, National Institutes of Health Stroke Scale; Mismatch volume, the difference between the ischemic penumbra volume and the core infarct volume; SHAP, SHapley additive explanations.

To enhance the interpretability of the RF prognostic model, the SHAP method was applied to explain model outputs at both the feature (global) and individual (local) levels. The SHAP summary plots (Figures 4a, b) illustrate the distribution and direction of each feature's contribution to the model output, with features ranked in descending order based on their mean absolute SHAP values. Admission NIHSS score and ischemic penumbra volume showed wide SHAP value distributions, suggesting strong predictive effects and a clear dose-response pattern. The SHAP dependence plots (Figure 5) further evaluated the effects of individual predictors on the RF output by illustrating how variations in feature values influence outcome predictions. A positive SHAP value greater than zero indicates that model's prediction shifts toward a poor functional prognosis. For example, WUS, hemorrhage transformation, and the presence of pneumonia were associated with positive SHAP values, thereby pushing predictions toward an unfavorable outcome. Similarly, when admission NIHSS score was ≥15, age ≥64 years, WBC count ≥7.8 × 10^9^/L, SBP ≥160 mmHg or ischemic penumbra volume ≥ 80 mL, the corresponding SHAP values were consistently higher than zero, indicating a higher likelihood of poor functional outcome.

**Figure 6:**
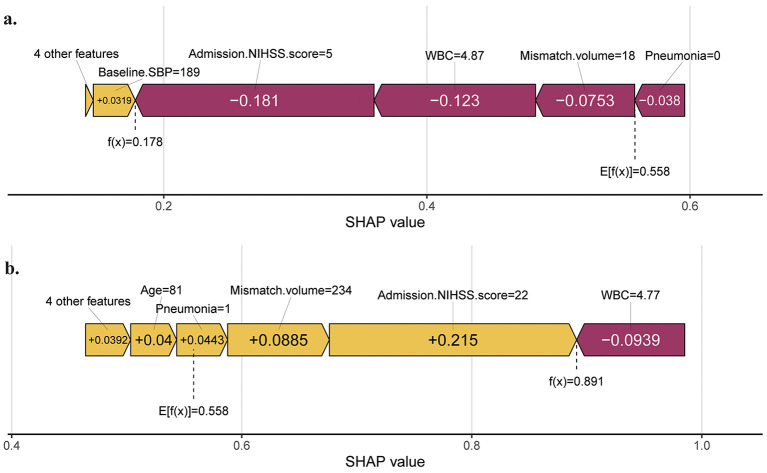
SHAP force plot analysis of feature contributions. **(a)** A representative patient with a low predicted probability of unfavorable prognosis (17.8%). **(b)** A representative patient with a high predicted probability of unfavorable prognosis (89.1%). Yellow bars represent features positively predicting an unfavorable prognosis, while red bars represent features negatively predicting an unfavorable prognosis. The length of each feature bar represents its relative contribution magnitude to model output. SBP, systolic blood pressure; NIHSS, National Institutes of Health Stroke Scale; WBC, white blood cell count; Mismatch volume, the difference between the ischemic penumbra volume and the core infarct volume; SHAP, SHapley Additive explanations.

At the patient level, the SHAP force plots in the validation set demonstrate how key features jointly contributed to individualized predictions. As shown in Figure 6, features with positive SHAP contributions push the prediction toward a poor functional outcome, whereas features with negative SHAP contributions are associated with a lower predicted risk. The length of each bar reflects the magnitude of that variable's contribution to the model's decision. In the case shown in Figure 6a, an admission SBP of 189 mmHg drove the prediction toward a poor outcome, whereas an admission NIHSS score of 5, a WBC count of 4.87 × 10^9^/L, an ischemic penumbra volume of 18 mL, and the absence of pneumonia during hospitalization acted as protective factors. The model predicted a 17.8% probability of poor prognosis for this patient. Figure 6b presents another case in which advanced age (81 years), a high admission NIHSS score (22), a large mismatch volume (234 mL), and the presence of pneumonia contributed to an increased predicted risk of poor functional outcome; among these factors, admission NIHSS score had the greatest influence.

## 7 Discussion

In this study, we developed models to predict poor functional outcomes in AIS patients receiving IVT in the extended time window using logistic regression and four ML algorithms. All models demonstrated excellent predictive performance, with AUC values above 0.80, indicating that both conventional logistic regression and advanced ML approaches achieved reliable predictive capability. The RF model achieved the highest AUC in the validation cohort, demonstrating superior overall performance. Considering the clinical need for early identification of high-risk patients, we specifically focused on model sensitivity as a key evaluation metric. Both the RF and XGBoost models maintained high sensitivity (>0.75) for identifying patients with poor outcomes. In direct comparisons of core performance metrics, the RF model significantly outperformed the others, with an accuracy of 0.73, a precision of 0.818, and an F1-score of 0.806. Collectively, these findings support RF as the optimal model in this cohort for predicting poor outcomes in AIS patients receiving IVT in the extended-time window.

Accurate prediction of short-term functional outcomes after ischemic stroke remains challenging, even for experienced neurologists. Using predictive models to predict functional outcomes is crucial for optimizing therapeutic strategies and guiding clinical decision-making. Previous ML-based studies have reported excellent performance in prognostic assessment for AIS patients receiving IVT, including prediction of early neurological deterioration, hemorrhage transformation and function outcomes (Li et al., 2025; Wang et al., 2020; Luo et al., 2025; Bu et al., 2025). However, most existing models have focused on patients treated within the conventional 4.5-h time window and commonly relied on clinical and laboratory variables alone. In contrast, our study integrated multimodal CT perfusion parameters with clinical and laboratory variables. Our RF model demonstrated high sensitivity and specificity for predicting poor functional outcomes, achieving an AUC of 0.854 in the validation cohort. These findings indicate that multimodal CT-based ML models can provide additional prognostic value for AIS patients receiving IVT beyond the standard therapeutic time window. By focusing on the extended therapy time window, our study addresses an important evidence gap and may better reflect real-world decision-making for patients who present beyond 4.5 h after stroke onset.

ML models are often criticized as “black-boxes” with limited interpretability, which can hinder clinical adoption. To overcome this limitation, we applied SHAP to enhance global and local interpretability. SHAP analysis of the RF model identified five key predictive features: admission NIHSS score, ischemic penumbra volume, pneumonia, WBC, and age. The admission NIHSS score reflects neurological deficits and assesses stroke severity, with higher scores indicating a greater likelihood of poor collateral circulation or concurrent large vessel occlusion (Kwah and Diong, 2014; Wardlaw et al., 2012; Heldner et al., 2013). Consistent with prior evidence, a higher NIHSS score was strongly associated with worse clinical prognosis in our models (Sadeghi-Hokmabadi et al., 2018; Kim D. H. et al., 2018; Zhao et al., 2017). The primary objective in AIS treatment is early and successful reperfusion of the ischemic penumbra, which is closely associated with collateral circulation (Goyal et al., 2015; Thomalla et al., 2020). Patients with robust collateral circulation typically develop smaller core infarct volumes and are more likely to achieve better clinical outcomes following IVT (Qian et al., 2020; Kim B. M. et al., 2018). In patients treated within an extended therapeutic time window, a larger ischemic penumbra volume often indicates more severe perfusion deficits and a higher of large vessel occlusion or incomplete reperfusion. Failure to achieve effective reperfusion in such cases may lead to unfavorable outcomes. In our study, bridging mechanical thrombectomy was administered to only 36.1% of eligible patients, while others did not undergo endovascular treatment due to factors such as advanced age, cost considerations, potential complications, or other clinical judgments. Consequently, despite having substantial ischemic penumbra volumes, failure to achieve reperfusion in AIS with large vessel occlusion likely contributed to poor clinical outcomes.

Pneumonia emerged as an important predictor, highlighting the impact of early post-stroke complications on patient recovery. Stroke-associated pneumonia typically occurs within the first week after stroke onset and is associated with worse functional outcomes and mortality (Eltringham et al., 2020; Huang et al., 2019). Some studies have demonstrated that pneumonia is an independent risk factor for poor prognosis in AIS patients, consistent with our findings (Teh et al., 2018; Hotter et al., 2021). Pneumonia increases the risk of secondary complications such as heart failure and myocardial infarction, often resulting in worse clinical outcomes (Cunha, 2001). Advanced age, pre-stroke mRS score, high admission NIHSS score, atrial fibrillation, dysphagia, and malnutrition are all high-risk factors for developing pneumonia after stroke (Yu et al., 2016; Feng et al., 2019; Li et al., 2023). Therefore, implementing rigorous pneumonia prevention strategies, including early recognition, prompt antibiotic treatment, and comprehensive care, is crucial for improving functional recovery in patients at high risk of poor outcomes.

We also included WUS patients, in whom the stroke onset time is unknown and is commonly estimated using the midpoint between the last known normal time and symptom recognition time (Ringleb et al., 2019). WUS account for approximately 20% of all AIS (Zhang et al., 2021). Owning to the uncertain onset, most WUS patients have been excluded from reperfusion therapies, only about 8–27% of WUS patients receive IVT, leading to neurological deterioration and poorer clinical outcomes (Manawadu et al., 2013). Our findings suggest that WUS patients undergoing IVT exhibit superior 90-day functional outcomes compared to standard acute ischemic stroke beyond the 4.5 h. The clinical or neuroimaging characteristics of WUS and morning-onset stroke patients were no significant differences, indicated that WUS attacked in a very short time before awakening, the time window of partial WUS patients may be at 3 h or less before awakening (Kidder et al., 2017; Kang et al., 2012). Accurate identification of symptom onset time is essential for thrombolytic treatment. A META-analysis of individual patient data in EXTEND, ECASS-4, and EPITHET trials demonstrated that among patients within 4.5–9 h of onset or WUS, IVT was associated with an increased risk of symptomatic intracranial hemorrhage but did not negate the overall net clinical benefits (Campbell et al., 2019). Therefore, the patients beyond 4.5 after stroke onset with perfusion mismatch can get favorable clinical outcomes through reperfusion therapy.

Although our study developed a high-performance predictive model using ML, several limitations should be acknowledged. First, this single-center retrospective study had a relatively small sample size, which may increase the risk of model overfitting and limit generalizability. Second, the absence of external validation represents a key limitation and further limits the generalizability of our findings; therefore, the model's reliability and broader applicability should be confirmed in independent, multi-center cohorts before clinical implementation. Third, we lacked serial assessments of the mRS scores at standardized post-thrombolysis intervals, precluding temporal analysis of dynamic prognostic risk factor evolution. To optimize model clinical utility, future studies should incorporate larger multicenter datasets, include longitudinal clinical measurements at various time pints, and perform external validation. From a methodological perspective, future research could benefit from integrating deep learning techniques. Specifically, convolutional neural networks could be used to achieve automatic segmentation of AIS lesions on non-contrast CT, multimodal CT and MRI images, to enable more detailed and standardized quantitative analysis of stroke-related imaging features (Wang et al., 2024). Combining deep learning-based imaging analysis with ML models applied to clinical and laboratory data could enhance predictive enhance predictive performance throughout the management of ischemic stroke, including risk prediction, diagnostic accuracy, and outcome assessment. Such integrated approaches can support more precise risk stratification, facilitate timely intervention for high-risk patients, and help reduce stroke-related disability and complications through earlier diagnosis and treatment. Moreover, they could provide clinicians and patients with more accurate predictions of functional outcomes, which can inform the development of individualized rehabilitation on strategies and long-term management plans.

## 8 Conclusion

We developed an explainable ML model that utilizes easily extractable clinical data from electronic medical records and imaging data to predict short-term functional outcomes in patients receiving low-dose alteplase IVT beyond the standard time window. In this single-center retrospective cohort, the RF model demonstrated favorable discrimination and calibration. However, due to the lack external validation, its generalizability and clinical applicability require confirmation in independent, multi-center cohorts. Pending external validation, the model has potential utility for risk stratification by identifying patients at higher predicted risk of poor functional outcomes, thereby informing early intervention and comprehensive assessment. Future prospective studies and large-scale clinical trials are required to determine whether model-guided, personalized, and timely interventions can improve functional outcomes.

## Data Availability

The raw data supporting the conclusions of this article will be made available by the authors, without undue reservation.
