# Supplementary material for: Development and validation of machine learning models for predicting functional outcome after low-dose alteplase in the extended time window for acute ischemic stroke
Source: Front Neurosci. 2026 May 8;20:1750031. doi: 10.3389/fnins.2026.1750031 (PMC13194576; doi:10.3389/fnins.2026.1750031)
Supplement: Supplementary file 1 [file Data_Sheet_1.pdf]

The ML codes of R software

```
library(mlr3)
library(mlr3verse)
library(mlr3extralearners)
mlr_learners
task <- as_task_classif(df, target = 'y')
set.seed(2026)
split = partition (task, ratio = 0.7)
```

Logistic Regression

```
at_log = lrn('classif.log_reg',predict_type = "prob")
at_log$train(task, row_ids = split$train)
predict_log_t = at_log$predict(task, row_ids = split$train)
predict_log_t$score(msr("classif.auc"))
predict_log_v = at_log$predict(task, row_ids = split$test)
predict_log_v$score(msr("classif.auc"))
```

LightGBM

```
lrn_lightgbm = lrn('classif.lightgbm',predict_type = "prob")
lrn_lightgbm$param_set
search_space_lightgbm = ps(
  max_depth = p_int(lower = 3, upper = 5),
  num_leaves = p_int(lower = 2, upper = 30),
  bagging_fraction = p_dbl(lower = 0.8, upper = 1),
  feature_fraction = p_dbl(lower = 0.8, upper = 1),
  lambda_l1 = p_int(lower = 0, upper = 1000),
  lambda_l2 = p_int(lower = 0, upper = 1000))
at_lightgbm = auto_tuner(
  tuner = tnr("grid_search",resolution = 10),
  learner = lrn_lightgbm,
```

```
  resampling = rsmp("cv", folds = 10L),
  measure = msr("classif.auc"),
  search_space = search_space_lightgbm,
  term_evals = 1000)
future::plan("multicore")
set.seed(2026)
predict_lightgbm_t = at_lightgbm$predict(task, row_ids = split$train)
predict_lightgbm_t$score(msr("classif.auc"))
predict_lightgbm_v = at_lightgbm$predict(task, row_ids = split$test)
predict_lightgbm_v$score(msr("classif.auc")
```

RandomForest

```
lrn_rf = lrn('classif.ranger',predict_type = "prob", importance = "impurity")
lrn_rf$param_set
search_space_rf = ps(
  num.trees = p_int(1, 20,trafo = function(x) 20 * x),
  mtry = p_int(2, 9),
  min.node.size = p_int(3, 30),
  max.depth = p_int(3, 9))
at_rf = auto_tuner(
  tuner = tnr("grid_search",resolution = 10),
  learner = lrn_rf,
  resampling = rsmp("cv", folds = 10),
  measure = msr("classif.auc"),
  search_space = search_space_rf,
  term_evals = 1000)
future::plan("multicore")
set.seed(2026)
at_rf$train(task, row_ids = split$train)
predict_rf_t = at_rf$predict(task, row_ids = split$train)
```

```
predict_rf_t$score(msr("classif.auc"))
predict_rf_v = at_rf$predict(task, row_ids = split$test)
predict_rf_v$score(msr("classif.auc"))
```

XGBoost

```
lrn_xgb = lrn('classif.xgboost',nrounds = 1000,
              predict_type = "prob",
              early_stopping_rounds = 1000,
              early_stopping_set = "test")
lrn_xgb$param_set
search_space_xgb = ps(
  eta = p_dbl(lower = 0.2, upper = 0.4),
  min_child_weight = p_dbl(lower = 1, upper = 20),
  subsample = p_dbl(lower = 0.7, upper = 0.8),
  colsample_bytree = p_dbl(lower = 0.9, upper = 1),
  colsample_bylevel = p_dbl(lower = 0.5, upper = 0.7),
  nrounds = p_int(lower = 1, upper = 25))#
at_xgb = auto_tuner(
  tuner = tnr("grid_search",resolution = 10),
  learner = lrn_xgb,
  resampling = rsmp("cv", folds = 10L),
  measure = msr("classif.auc"),
  search_space = search_space_xgb,
  term_evals = 1000)
future::plan("multicore")
set.seed(2026)
predict_xgb_t = at_xgb$predict(task, row_ids = split$train)
predict_xgb_t$score(msr("classif.auc"))
predict_xgb_v = at_xgb$predict(task, row_ids = split$test)
predict_xgb_v$score(msr("classif.auc"))
```

SVM

```
lrn_svm = lrn("classif.svm", type = "C-classification",predict_type = "prob")
lrn_svm$param_set
search_space_svm = ps(
  cost = p_dbl(lower = 0.1, upper = 10),
  gamma = p_dbl(lower = 0, upper = 5),
  kernel = p_fct(c("polynomial", "radial")))
at_svm = auto_tuner(
  tuner = tnr("grid_search",resolution = 10),
  learner = lrn_svm,
  resampling = rsmp("cv", folds = 10),
  measure = msr("classif.auc"),
  search_space = search_space_svm,
  term_evals = 1000)
set.seed(2026)
predict_svm_t = at_svm$predict(task, row_ids = split$train)
predict_svm_t$score(msr("classif.auc"))
predict_svm_v = at_svm$predict(task, row_ids = split$test)
predict_svm_v$score(msr("classif.auc"))
```
