# Supplementary material for: Development and validation of machine learning models for predicting functional outcome after low-dose alteplase in the extended time window for acute ischemic stroke
Source: Front Neurosci. 2026 May 8;20:1750031. doi: 10.3389/fnins.2026.1750031 (PMC13194576; doi:10.3389/fnins.2026.1750031)

**Development and Validation of Machine Learning Models for Predicting Functional Outcome after Low-Dose Alteplase in the Extended Time Window for Acute Ischemic Stroke**

**Table legends**

Table S1: Summary of patient characteristics and variables.

Table S2: Comparison of performance of ML and Logistic Regression in the training cohort.

**Figure legends**

Figure S1: Penalty chart of predictive factors based on LASSO regression analysis.

Figure S2: LightGBM models confusion matrix in the training sets (a) and validation sets (b).

Figure S3: RF models confusion matrix in the training sets (a) and validation sets (b).

Figure S4: Logistic regression models confusion matrix in the training sets (a) and validation sets (b).

Figure S5: SVM models confusion matrix in the training sets (a) and validation sets (b).

Figure S6: XGBoost models confusion matrix in the training sets (a) and validation sets (b).

Figure S7: Five-fold and ten-fold cross-validation of the final RF model in the validation set.

Figure S8: Calibration curves of five ML models in the training sets (a) and validation sets (b).

Figure S9: DCA curves of five ML models in the training sets.

**Table S1. Summary of patient characteristics and variables**

| **Category** | **Variables** |
| --- | --- |
| Demographic Factors | Age  Sex |
| Risk factors | Hypertension  Diabetes mellitus  Hyperlipidemia  Atrial fibrillation  Previous stroke  Prior antiplatelet or anticoagulant therapy |
| Clinical characteristics | Baseline systolic/diastolic blood pressure  Admission NIHSS score  Wake-up stroke  Stroke onset-to-door time  Door-to-needle time  Mechanical thrombectomy |
| Laboratory tests | Fasting blood glucose  Hemoglobin A1c  White blood cell count  C-reactive protein  Platelet count  Hemoglobin  Prothrombin time  Activated partial thromboplastin time  Total cholesterol  Triglycerides  Low-density lipoprotein cholesterol  High-density lipoprotein cholesterol  Homocysteine  Blood urea nitrogen  Serum creatinine |
| Admission Imaging data based on CT | Alberta Stroke Program Early CT Score  Core infarct volume  Ischemic penumbra volume  Mismatch volume |
| MRI-determined infarct-associated culprit vessels | Anterior cerebral artery  Middle cerebral artery  Posterior cerebral artery  Vertebral-basilar artery  Internal cerebral artery |
| TOAST classification | Large-artery atherosclerosis  Cardioembolism  Small-artery occlusion  Stroke of other determined etiology  Stroke of undetermined etiology |
| Safety Outcomes | Any intracranial hemorrhage  Hemorrhage transformation  Symptomatic intracranial hemorrhage  Pneumonia |

**Table S2** Comparison of performance of machine learning and Logistic Regression in the training cohort

| Models | AUC | Sensitivity | Specificity | Accuracy | PPV | NPV | F1-score | Brier score |
| --- | --- | --- | --- | --- | --- | --- | --- | --- |
| SVM | 0.970(0.942-0.998) | 0.951 | 0.987 | 0.925 | 0.987 | 0.910 | 0.984 | 0.106 |
| RF | 0.951(0.921-0.982) | 0.887 | 0.872 | 0.938 | 0.872 | 0.911 | 0.823 | 0.113 |
| Light GBM | 0.861(0.803-0.919) | 0.761 | 0.897 | 0.650 | 0.897 | 0.667 | 0.903 | 0.155 |
| Logistic Regression | 0.838(0.775-0.901) | 0.739 | 0.877 | 0.625 | 0.877 | 0.647 | 0.887 | 0.165 |
| XGBoost | 0.753(0.673-0.833) | 0.718 | 0.700 | 0.875 | 0.700 | 0.762 | 0.516 | 0.218 |

AUC, Area Under the Curve; SVM, Support Vector Machine; RF, Random Forest; Light GBM, Light Gradient Boosting Machine;

XGBoost, Extreme Gradient Boosting; PPV, positive predictive value; NPV, negative predictive value.

**Figure S1** Penalty chart of predictive factors based on LASSO regression analysis


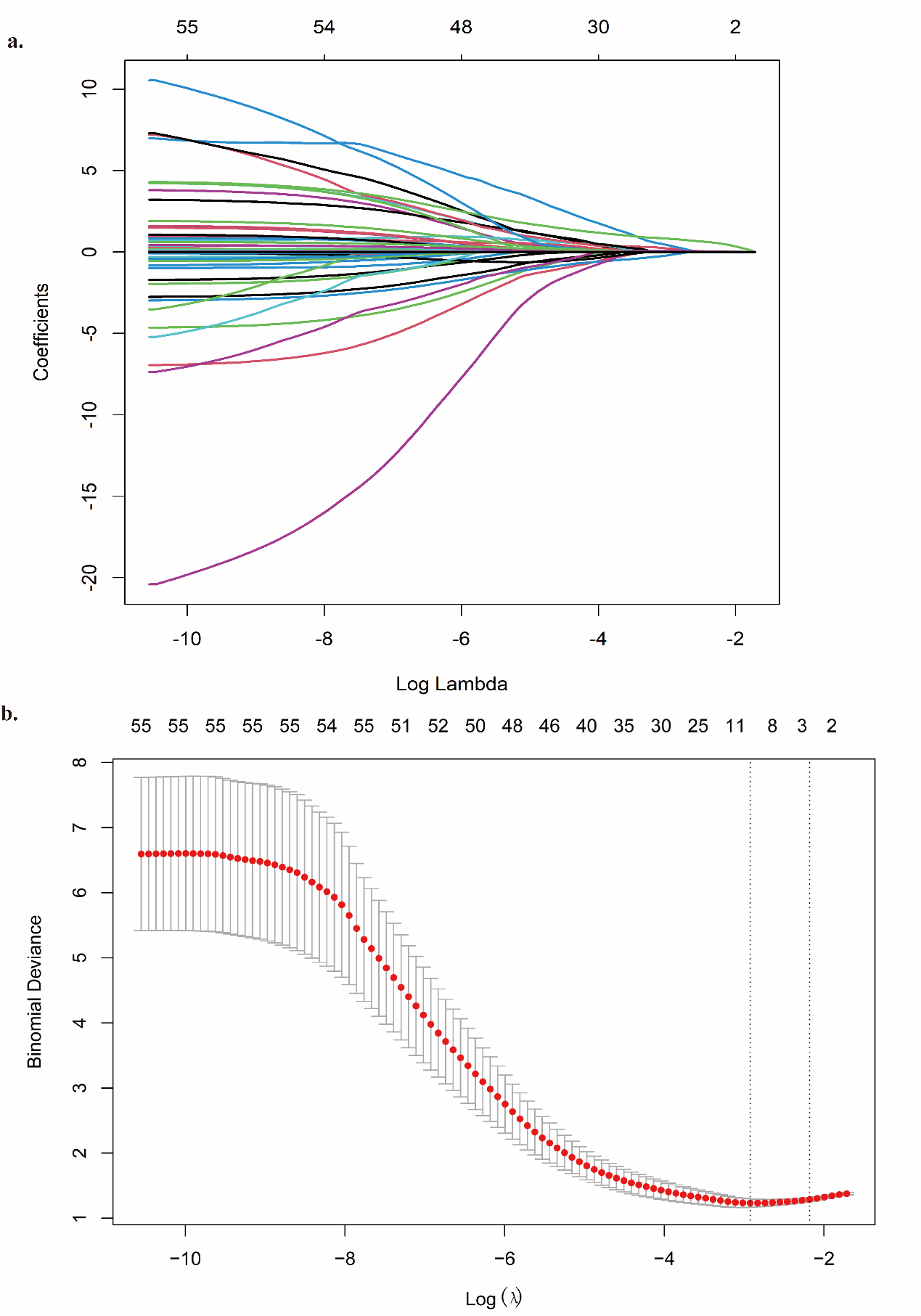


a. LASSO coefficient paths across regularization parameters; b. LASSO coefficient paths across regularization parameters (λ).

**Figure S2** LightGBM models confusion matrix in the training sets (a) and validation sets (b)


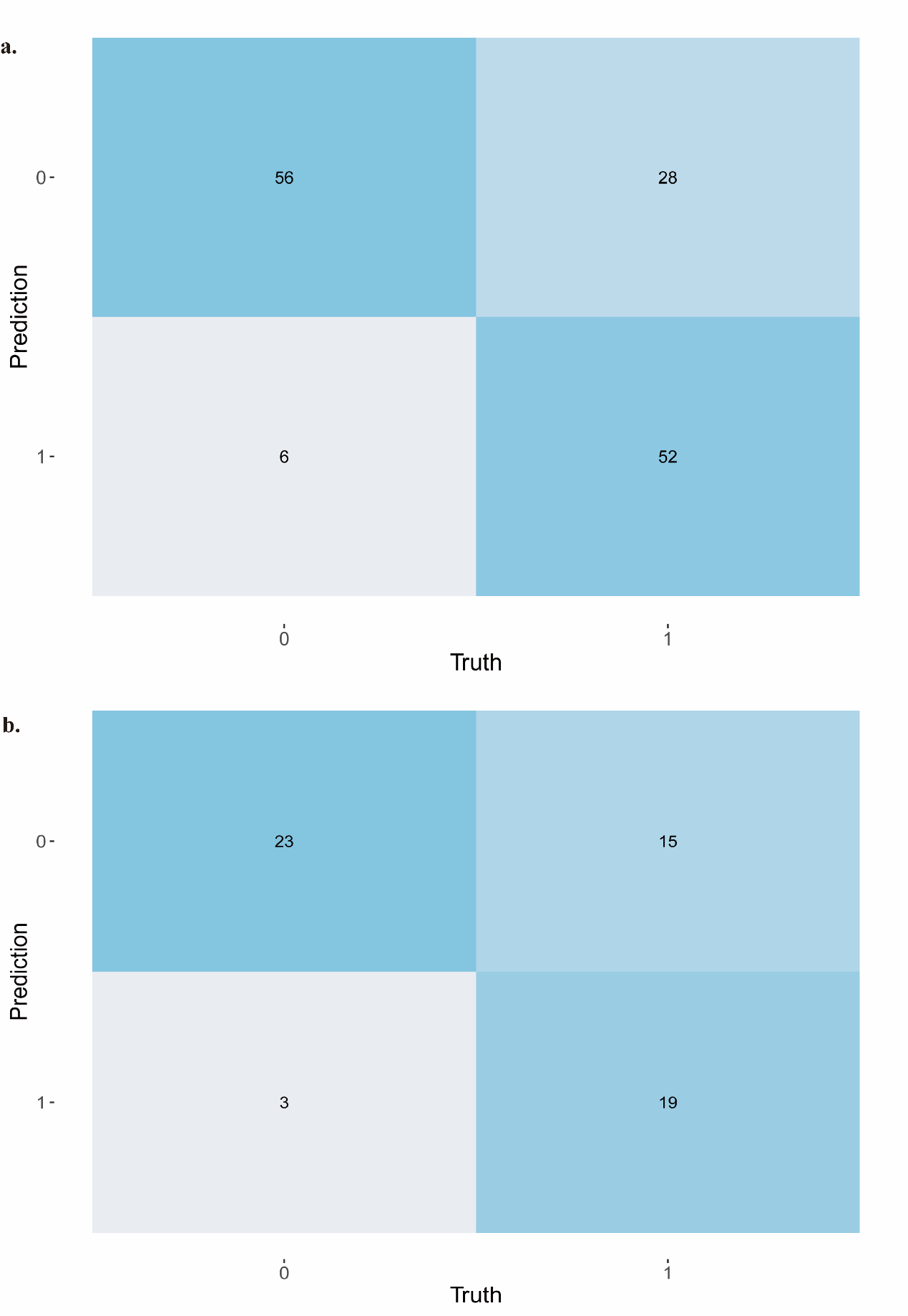


**Figure S3** RF models confusion matrix in the training sets (a) and validation sets (b)


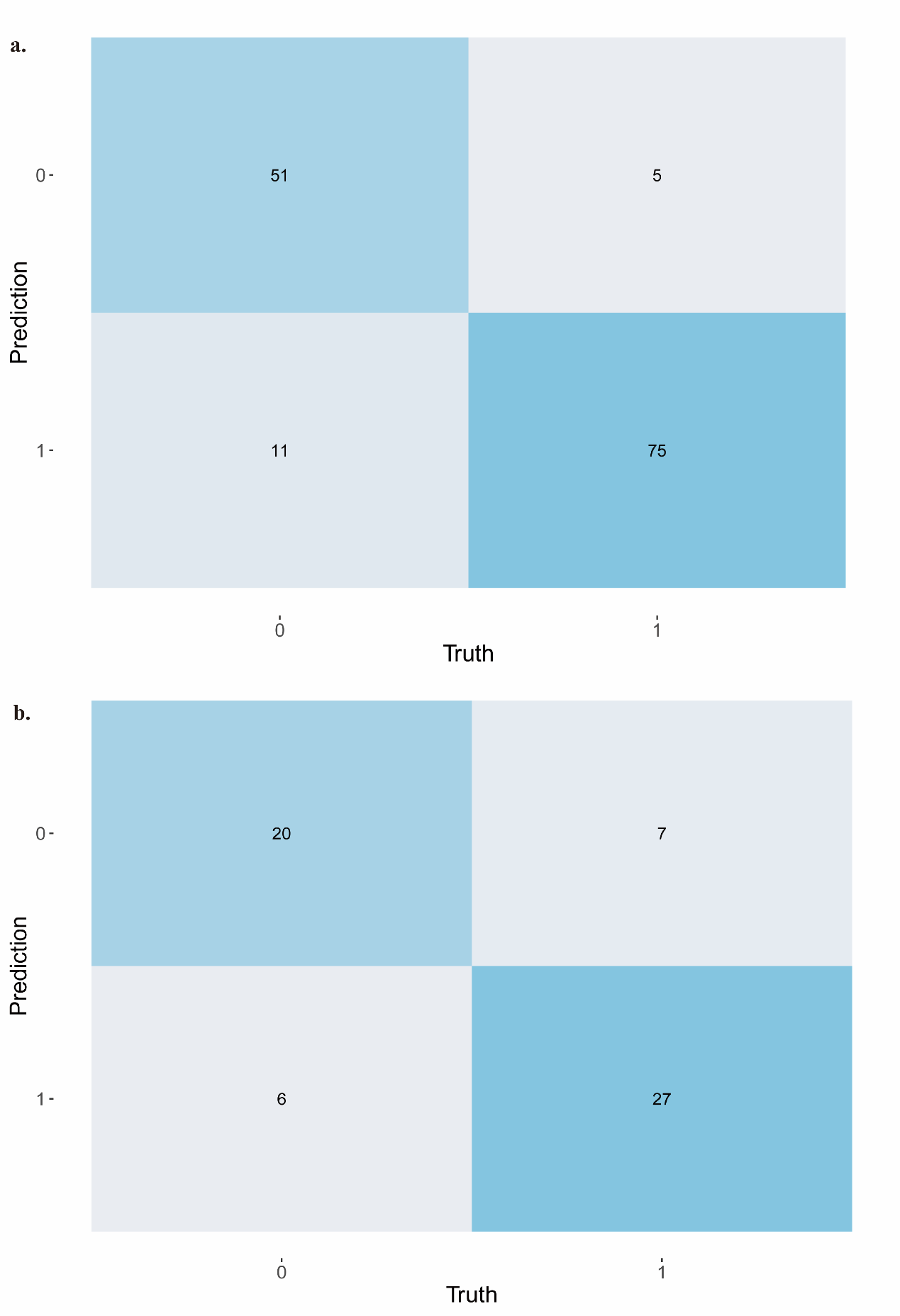


**Figure S4** Logistic regression models confusion matrix in the training sets (a) and validation sets (b)


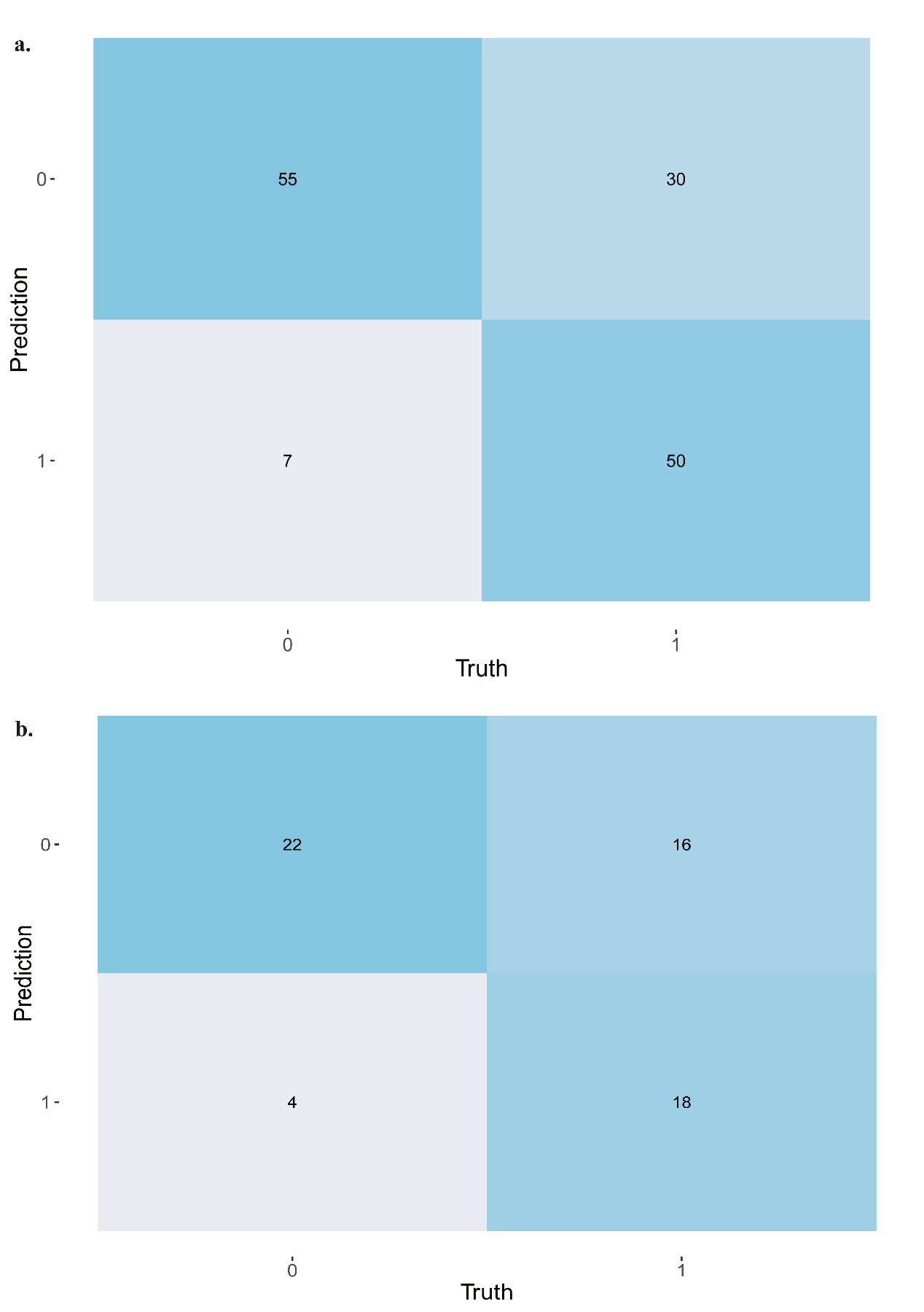


**Figure S5** SVM models confusion matrix in the training sets (a) and validation sets (b)


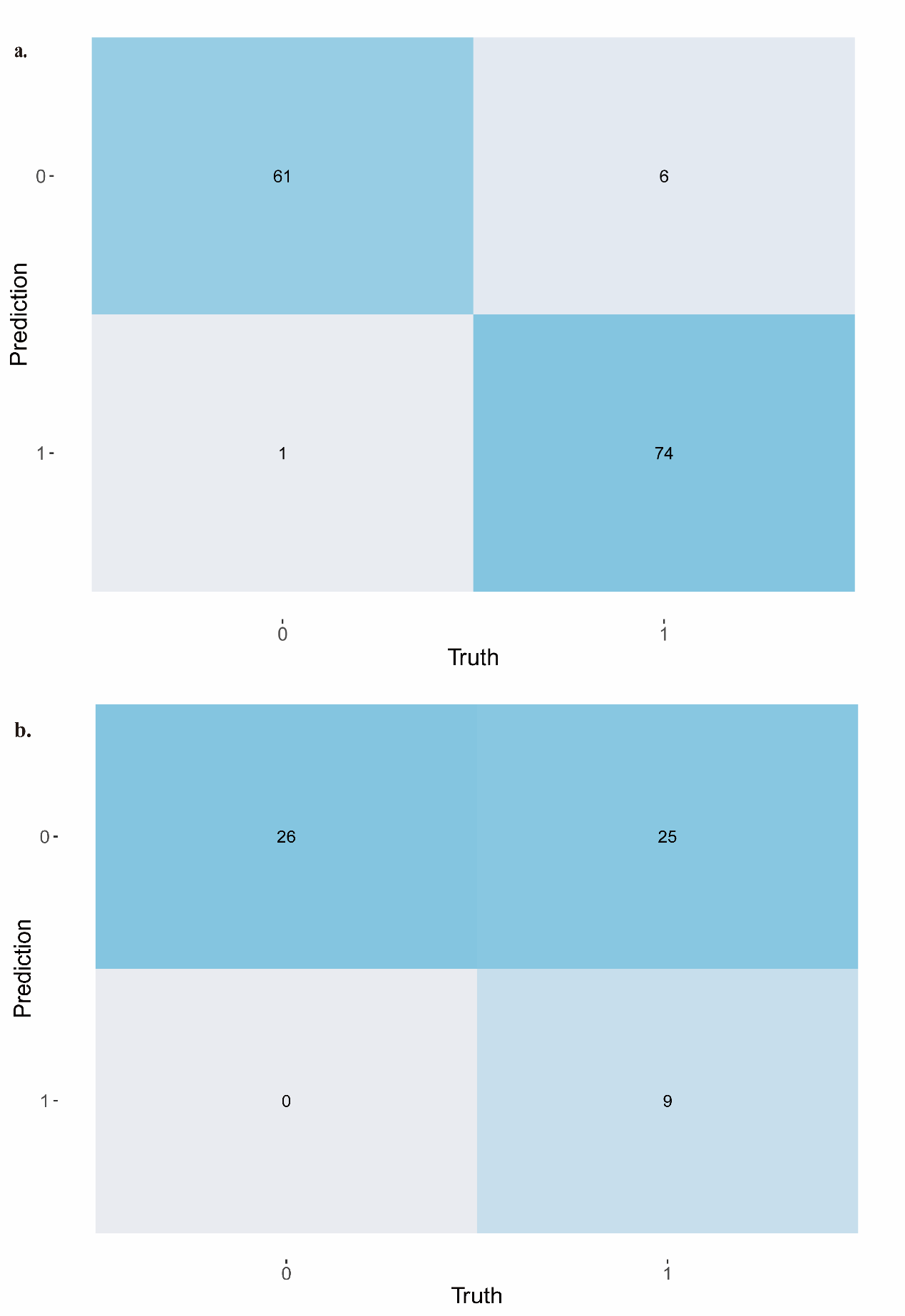


**Figure S6** XGBoost models confusion matrix in the training sets (a) and validation sets (b)


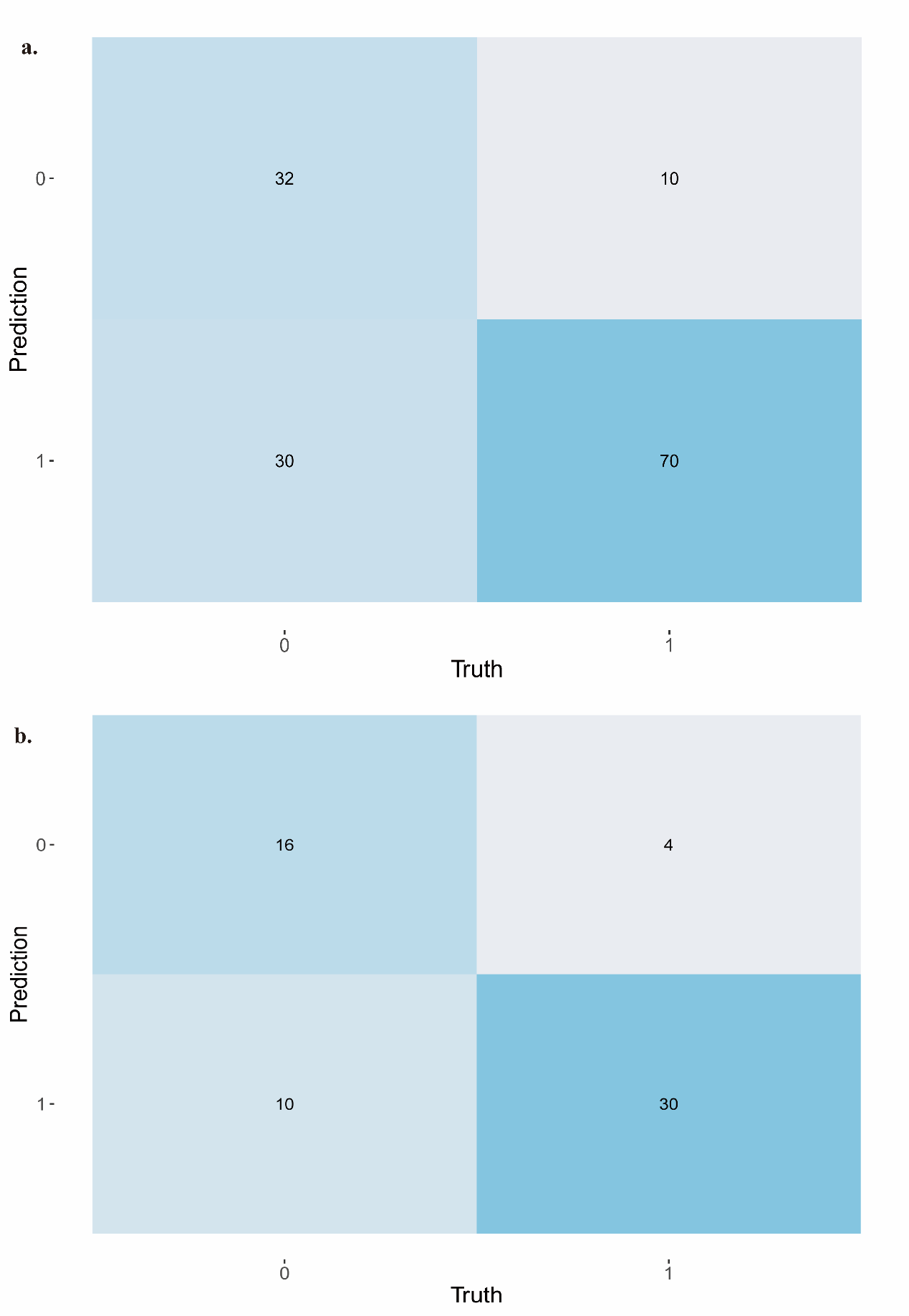


**Figure S7** Five-fold and ten-fold cross-validation of the final RF model in the validation set

**
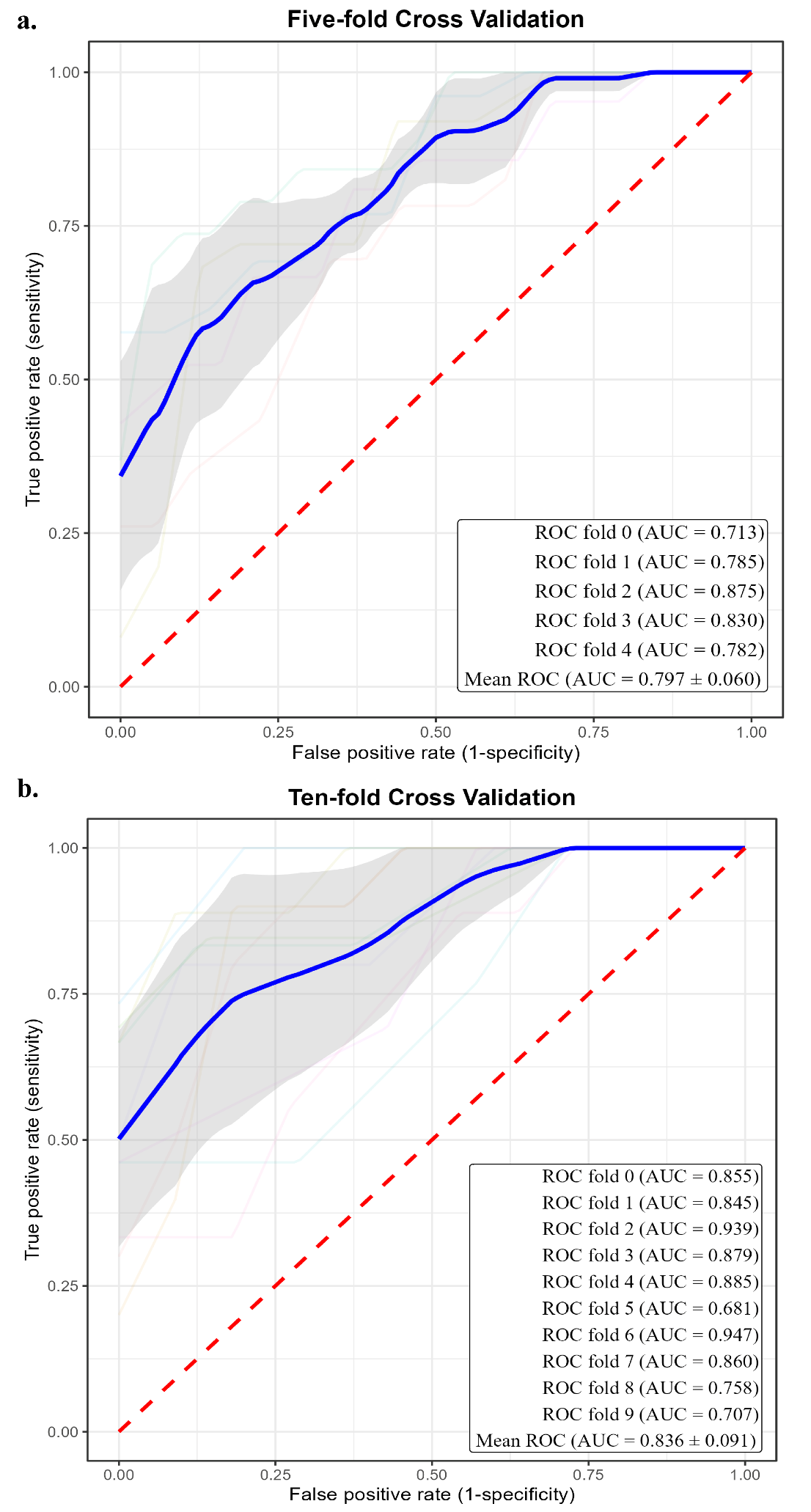
**

**Figure S8** Calibration curves of five ML models in the training sets (a) and validation sets (b)


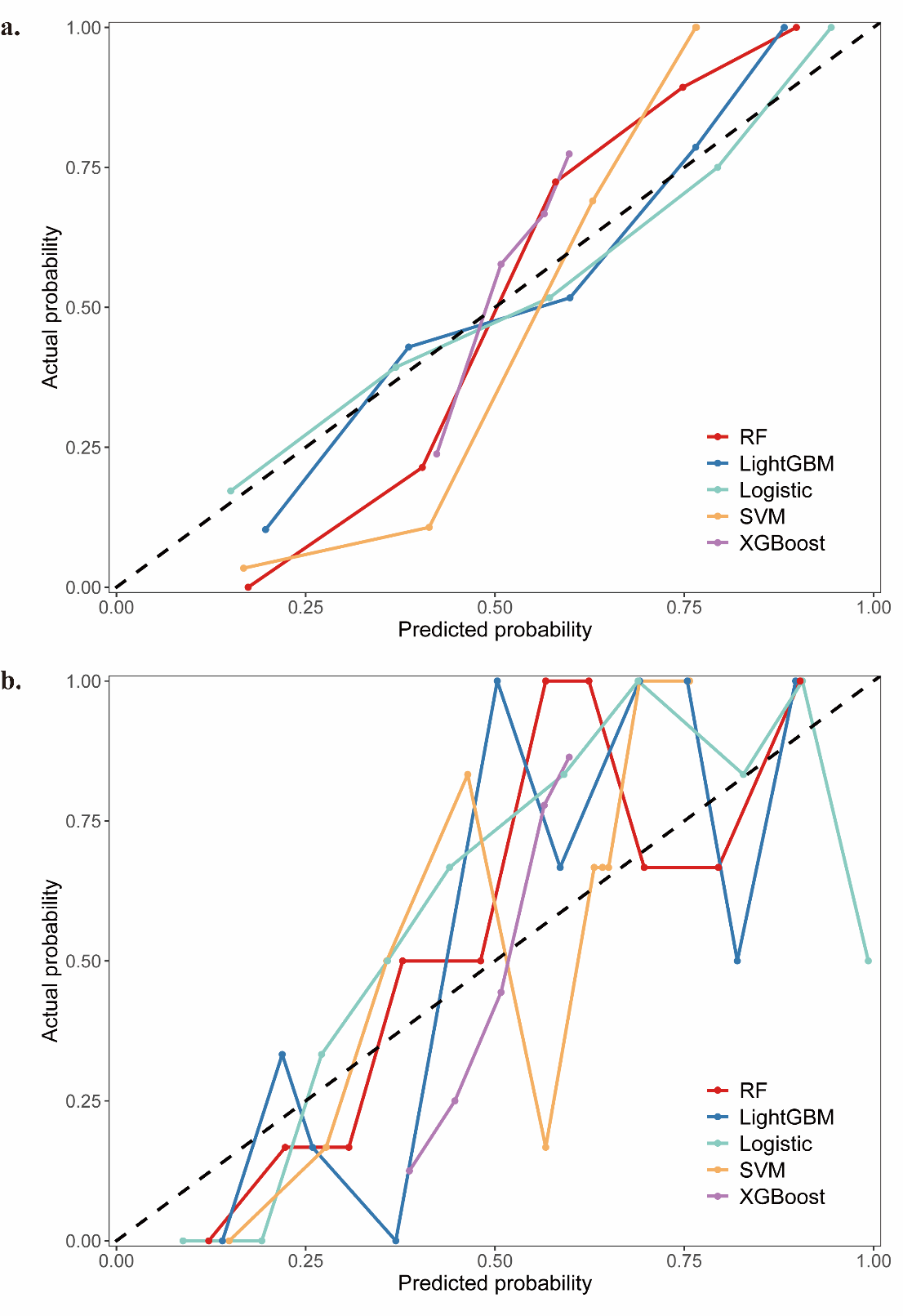


**Figure S9** DCA curves of five ML models in the training sets


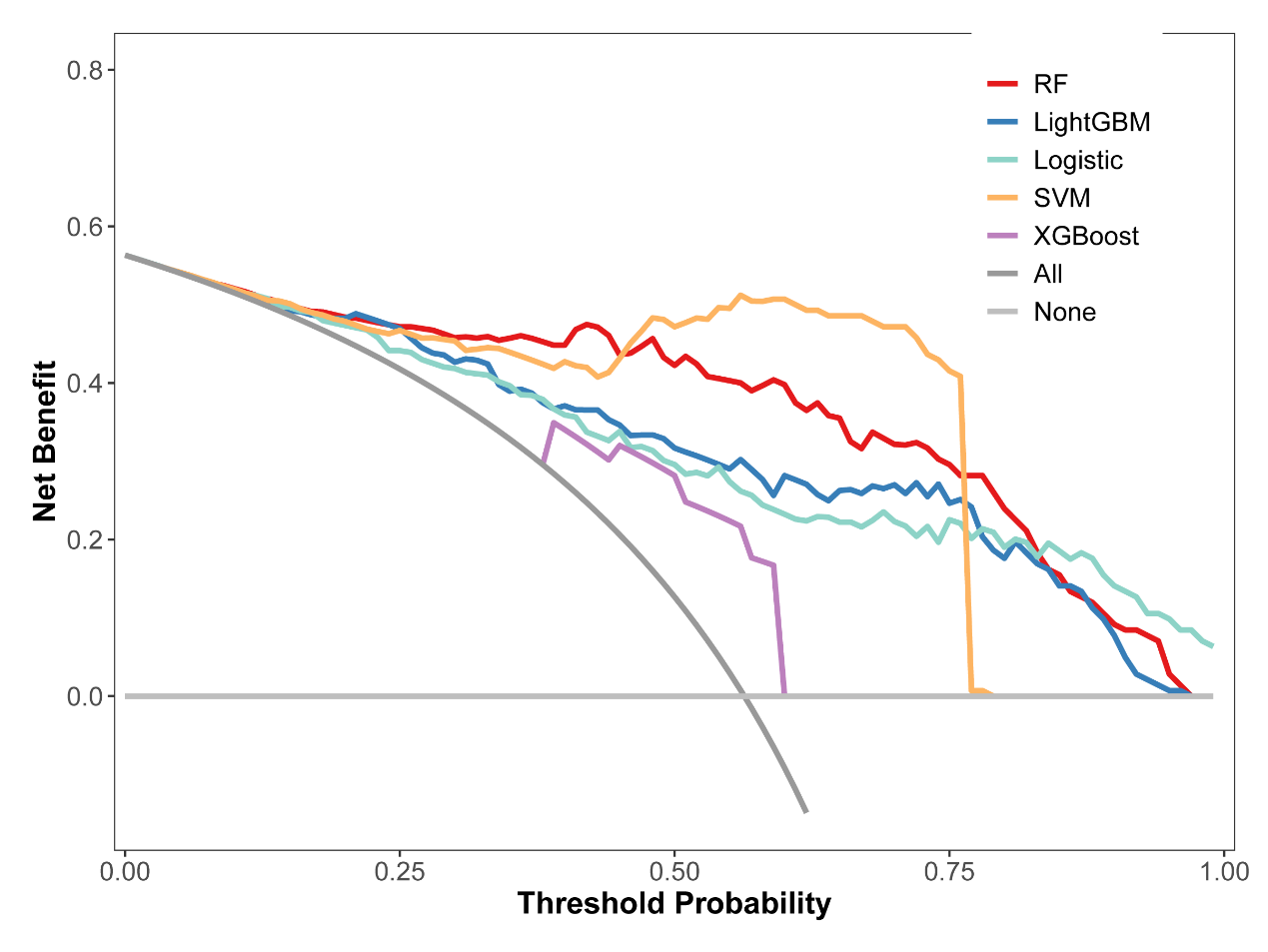

Supplement: Supplementary file 2 [file Data_Sheet_2.docx]
